# Chia Oil Adulteration Detection Based on Spectroscopic Measurements

**DOI:** 10.3390/foods10081798

**Published:** 2021-08-04

**Authors:** Monica Mburu, Clement Komu, Olivier Paquet-Durand, Bernd Hitzmann, Viktoria Zettel

**Affiliations:** 1 Institute of Food Bioresources Technology, Dedan Kimathi University of Technology, Private Bag, Dedan Kimathi, Nyeri 10143, Kenya; monica.mburu@dkut.ac.ke (M.M.); clementmumo@gmail.com (C.K.); 2 Department of Process Analytics and Cereal Science, Institute of Food Science and Biotechnology, University of Hohenheim, Garbenstr. 23, 70599 Stuttgart, Germany; O.Paquet-Durand@uni-hohenheim.de (O.P.-D.); Bernd.Hitzmann@uni-hohenheim.de (B.H.)

**Keywords:** chia oil, adulteration, spectroscopy, NIR, Raman, fluorescence

## Abstract

Chia oil is a valuable source of omega-3-fatty acids and other nutritional components. However, it is expensive to produce and can therefore be easily adulterated with cheaper oils to improve the profit margins. Spectroscopic methods are becoming more and more common in food fraud detection. The aim of this study was to answer following questions: Is it possible to detect chia oil adulteration by spectroscopic analysis of the oils? Is it possible to identify the adulteration oil? Is it possible to determine the amount of adulteration? Two chia oils from local markets were adulterated with three common food oils, including sunflower, rapeseed and corn oil. Subsequently, six chia oils obtained from different sites in Kenya were adulterated with sunflower oil to check the results. Raman, NIR and fluorescence spectroscopy were applied for the analysis. It was possible to detect the amount of adulterated oils by spectroscopic analysis, with a minimum R^2^ of 0.95 for the used partial least square regression with a maximum RMSEP_range_ of 10%. The adulterations of chia oils by rapeseed, sunflower and corn oil were identified by classification with a median true positive rate of 90%. The training accuracies, sensitivity and specificity of the classifications were over 90%. Chia oil B was easier to detect. The adulterated samples were identified with a precision of 97%. All of the classification methods show good results, however SVM were the best. The identification of the adulteration oil was possible; less than 5% of the adulteration oils were difficult to detect. In summary, spectroscopic analysis of chia oils might be a useful tool to identify adulterations.

## 1. Introduction

Chia, *Salvia hispanica* L., a member of the Labiatae family, is cultivated in environments ranging from tropical to subtropical conditions and used as a food ingredient. Native from southern Mexico and northern Guatemala, chia has been cultivated on a commercial basis in Australia, Colombia, Argentina, Peru, Ecuador, Bolivia and Paraguay [1]. Research has proved that chia seeds are a good source of oil, protein, dietary fiber, minerals and polyphenolic compounds [2]. Quantitatively, chia seeds contain 91–93 g/100 g dry matter, 26–41 g/100 g carbohydrates, 32–39 g/100 g oil, 22–24 g/100 g protein, 18–30 g/100 g dietary fiber, and 4–6 g/100 g ash, vitamins, antioxidants, minerals contents [3].

Chia oil is known to lower the risks of cardiovascular disease, inflammation, hepatoprotective effect and also to prevent the likelihood of obesity-related disorders [4]. According to research carried out by Gazem et al. [5], investigating in vitro the cancer cytotoxic properties of chia seeds oil and its blends, chia seed oil was found to significantly inhibit anti-lipoxygenase activity, and demonstrated potent and differential anticancer activity. The team concluded that supplementation of a modern diet with chia seeds oil may delay or prevent the incidence of degenerative disorders. Additionally, according to research carried out by Albert et al. [6], it was observed that supplementation of a diet with long-chain omega-3 polyunsaturated fatty acids can prevent cardiovascular and inflammatory diseases. Current research has not shown any adverse effects of chia seed consumption, but toxicological data on controlled human trials on the safety and efficacy of chia seed oils are still limited. With the emerging concepts around the combination of chemotherapy and nutritional therapy, there is need to increase data on fatty acid composition in various foods that can be applied in chemotherapeutic subjects. Chia seed oil is becoming an appealing and preferred choice for healthy food and cosmetic applications due to its lower content of saturated fatty acids (palmitic and stearic acids) and adequate concentration of linolenic fatty acids (55–60%) and linoleic acids (18–20%) [3]. Both chia seeds and chia seed oil have been safely applied in animal feeds to decrease the cholesterol levels and increase the polyunsaturated fatty acids and in egg and meat products [7]

Extraction of chia oils apply different methods with diverse oil yields including cold-pressing followed by centrifugation to remove physical matter, hot-pressing, solvent extraction and supercritical fluid. Chia oil yield and quality in terms of fatty acids composition are affected by several factors including agroecological zones of growth, seed variety, seed storage conditions, pre-treatment method, size reduction practices and the aforementioned extraction procedures [8]. Due to the high value of chia oil, some unscrupulous sellers may adulterate with cheaper oils in order to increase profit. This adulteration will also make the long-chain polyunsaturated fatty acids highly susceptible to lipid hydrolysis and oxidation, thus loosing shelf-life, consumer acceptability, nutritional value, functionality and safety.

Vegetable oils are valuable component of human nutrition. Adulteration of valuable expensive oils with cheaper oils is very common practice. Applying spectroscopic methods provides an opportunity quickly detect these adulterations. There are several works available on olive oil adulteration detection by fluorescence spectroscopy [9,10,11]. Sikorska et al. [12] were able to distinguish between different edible oils using fluorescence spectroscopy. Near Infrared spectroscopy (NIR) is also well established for food analysis [13]. With data obtained from NIR, UV-Vis and GC, the ComDim chemometrics method was able to distinguish 32 vegetable oil samples by their characteristics and compositions [14]. Rodríguez et al. [15] showed that it is possible to detect adulteration of sesame and chia oils by Fourier transform infrared spectroscopy with prediction errors between 1% and 5%. Studies on oil adulteration detection with spectroscopic methods have been published by several authors. For example, La Mata et al. [16] used ATR-FTIR spectroscopy and were able to differentiate between blends with olive oil content higher than 50% (*w/w*) and those below 50% (*w/w*). More examples for the application of FTIR on olive oil adulteration can be found in literature [17,18,19,20]. (FT- or M-) IR spectroscopy was also successfully used for sesame oil adulteration [21,22,23,24,25]. Extra virgin olive oil adulteration with hazelnut oil was evaluated using mid-infrared and Raman spectroscopic data [26]. The application of Raman spectroscopy on olive oil adulteration [27] or the combination of Raman and NIR spectroscopy [28] is another way of combining the spectroscopic methods. Adulteration detection by FT-Raman and NIR spectroscopy, combined with data fusion and Soft Independent Modelling of Class Analogy, was performed on a case study to determine the adulteration of hazelnut paste with almonds or chickpeas [29]. Other examples of combinations of NIR and fluorescence were given by Hu et al. [30], who worked on the fraud detection of Chinese tea oil or by Li et al. [31], who applied these spectroscopic methods to detect adulteration and authenticity of walnut oil.

This study focuses on the adulteration of chia oils with cheaper oils that are available in European and African markets. The more expensive chia oils are currently paid a great deal of attention in African countries, and therefore it is necessary to prevent the valuable oil from adulteration. Adulteration detection is mostly dependent on discriminant analysis, where the spectrum of the test sample is compared to a reference library. The establishment of the reference library usually takes a long time due to the amount of data that has to be covered, e.g., known adulterated samples. Important questions must be answered throughout the process, such as whether a test sample belongs to the native samples or the adulterated samples and whether the adulteration can actually be identified. The last but most difficult question is to which amount the test sample has been adulterated.

## 2. Materials and Methods

### 2.1. Sample Preparation

Two different samples of chia oil were purchased, A: Bio Chia Öl (Ölmühle Fandler GmbH, Pöllau, Austria with best before dates of 21 January 2020 and 28 February 2020, origin: Mexico) and B: Chiaöl (Ölmühle Solling GmbH, Boffzen, Germany with best before dates of 7 September 2019 and 26 December 2019, origin: Mexico). For adulteration, common food preparation oils were purchased at the local markets: rapeseed oil (R): Reines Rapsöl, raffiniert (Bökelmann + Co. Ölmühle GmbH & Co. KG, Hamm, Germany, with best before date 24 April 2020), sunflower oil (S): Reines Sonnenblumenöl, raffiniert (Walter Rau Lebensmittelwerke GmbH, Hilter, Germany, with best before date 17 May 2020), and corn oil (C): Mazola, reines Maiskeimöl (Peter Kölln GmbH & Co. KG, Elmsholm, Germany, with best before date 27 May 2020). The nutritional values of the oil samples are presented in Table 1. 

In Table 2, the sample preparation and its labelling for the Mexican chia oils is presented. Every sample was prepared three times, and 114 samples were collected. The sample volume remained constant at 3.5 mL.

For Kenyan chia oil samples, named oil U, V, W, X, Y, Z (from chia seeds obtained from different growth sites in Kenya) a smaller sample volume (2 mL) was chosen because of the small number of samples available. Its samples were prepared, according to Table 3, two times with exceptions (indicated with *), which were prepared once. Therefore, 28 different samples were obtained from Kenyan chia oil. All samples were directly prepared in a quartz glass cuvette and mixed by gently shaking. Then the cuvettes were placed in the respective spectrometer.

### 2.2. Spectroscopic Measurements

Three spectrometers were used to obtain near infrared (NIR), Raman and fluorescence spectra of the oil samples. NIR spectroscopy measurements were performed in the Multi-Purpose NIR Analyzer (Bruker Optik GmbH, Ettlingen, Germany), varying wavelengths from 800 nm to 2800 nm, in absorbance, with a resolution of 15 nm and 8 scans per measurement.

Raman spectroscopy was performed with a FT-Raman785 spectrometer (Inno-spec GmbH, Model 11-0130005-119, Nürnberg, Germany), equipped with a 784.98 nm Laser applying a measurement range from 350 cm^−1^ to 3200 cm^−1^. The integration time was 1 s and 3 scans were performed for each measurement. The background was measured with an empty cuvette.

3D-fluorescence spectra were obtained with FluoroMax4 Spectrofluorometer (HORIBA JOBIN YVON Technology, Edison, NY, USA). Spectra were analysed in a range between 300 nm and 550 nm of excitation and 350 nm and 700 nm emission with 10 nm distance steps and a slit width of 1 nm. In total, the resulting spectra contained 936 measured intensities of wavenumber and wavelength combinations.

Every prepared sample was measured 5 times. In total, 142 samples were measured. Every single spectrum was used for the analysis, in total 710 spectra were obtained for each spectroscopic method. The resulting combined spectra contained 2751 points.

### 2.3. Spectra Evaluation: Preprocessing

The evaluation of the spectra was performed with Matlab R2020a (version 9.8). The spectra were pre-processed with different methods to extract the desired information. A baseline correction and a standard normal variate (SNV) transformation was applied to Raman and NIR spectra. For the baseline correction, the following Matlab code, presented in Equation (1), was applied in a loop using the intensity values of all wavenumbers k in a spectrum.

(1) $$I_{BC}\left(k\right)=I\left(k\right)-cumsum\left[smooth\left(diff\left\{I\left(k\right)\right\},20\right)\right]$$

$I_{BC}\left(k\right)$ is the baseline corrected intensity value, I(k) the raw intensity, cumsum, smooth and diff are Matlab functions. To harmonize the spectra further, a standard normal variate transformation, presented in Equation (2), was applied as follows

(2) $$I_{SNV}\left(k\right)=\frac{I_{BC}\left(k\right)-{\bar{I}}_{BC}}{SD_{BC}}$$

$I_{SNV}\left(k\right)$ is the transformed intensity, ${\bar{I}}_{BC}$ and $SD_{BC}$ are the mean value and standard deviation of the base line corrected spectrum. For the fluorescence spectra, no pre-processing was applied. The spectra were then evaluated separately for each spectrometer typ. For further evaluations NIR, fluorescence and Raman spectra were combined. The intensities of the fluorescence spectra were therefore scaled down with a SNV transformation, subsequently the NIR and Raman spectra were appended to the fluorescence spectra to produce combined spectra.

### 2.4. Spectra Evaluation: Classification

The classification was performed by using the Classification Learner App, which is implemented in Matlab. The following classification algorithms were tested: decision tree (DT), linear discriminant analysis (LD), k nearest neighbour classification (KNN), support vector machine linear (SVMl) and cubic (SVMc). The classification was performed with 5 classes: 1. A, 2. Adult A, 3. B, 4. Adult B, 5. Adult. The classification was performed to check if A and B samples of the native oils could be distinguished and if an adulteration was present. For Adult A and Adult B, the 12 samples with A and B were complemented by 3 corresponding samples of the additional combinations presented in Table 2. Therefore, 225 spectra were in both classes. To obtain equal number of spectra in every class some simulation spectra were calculated, so that every class was enlarged to 225 spectra.

The number of pure oil samples of class A and B resulted just in 15 spectra each, therefore new spectra were simulated out of them. First, the means m and the standard deviations SD of intensity values for all wavenumbers (Raman and NIR) or wavelength combination (fluorescence) for both classes were individually calculated. 150 spectra for each pure oil sample were simulated by adding to each value in the mean (75) or the original (75) spectrum the corresponding standard deviation times a standard normal distributed random number, which has a cero mean and a standard deviation of one, as shown in Equation (3).

(3) $$\tilde{I}\left(k\right)=I\left(k\right)+SD\left(k\right)\times ran\left(k\right)$$

Here $\tilde{I}\left(k\right)$ is the simulated intensity value, k is either the wavenumber (for Raman and NIR) or an index for the wavelength combinations (fluorescence), I(k) is the corresponding mean or original value and SD(k) the corresponding standard deviation, ran(k) is a standard normal distributed random number, which is calculated for each k. To complete the class A and B data sets to 225 spectra, the original spectra were used five times.

For the “Adult” class, the 100% pure samples of S, R and C as well as the corresponding additional combinations (RS50, RC50, SC50), which were 90 spectra together, were complemented by 90 simulated spectra obtained in the same manner as discussed before (Equation (3)) from the samples S, R and C. To complete the data set of class “Adult”, 45 replication spectra from S, R and C samples were added. In total 1125 spectra were obtained, where each class consisted of 225 spectra.

The quality of the classification is assessed with the amount of correct detected samples, which is calculated as % of samples in the validation dataset and is presented as True Positive Rate (TPR). The sensitivity (Equation (4)), specificity (Equation (5)), accuracy (Equation (6)) and precision (Equation (7)) are calculated with the values of true positive (TP), true negative (TN), false negative (FN) and false positive (FP) identified samples [32].

Sensitivity (%) = TP/(TP+FN)∙100%,(4)

Specificity (%) = TN/(TN+FP)∙100%,(5)

Accuracy (%) = (TP+TN)/(TP+TN+FP+FN)∙100%,(6)

Precision (%) = TP/(TP+FP) 100%,(7)

### 2.5. Spectra Evaluation: Partial Least Squares Regression

Partial Least Squares Regression (PLSR) models are calculated for each oil to predict the adulteration levels. For the Mexican chia oil samples, A and B, 1 up to 32 principal components (3–10 for Kenyan samples, depending on the number of measured samples) are tested for the PLSR model. A leave-one-out-cross-validation (CV) is performed for each dataset. The coefficient of determination R^2^ and the root mean square error of prediction RMSEP_range_ are calculated.

The detection limit dl for the PLSR was calculated from the blank sample (100% pure chia oil) with Equation (8), where m is the mean and SD is the standard deviation.

dl = m_100% chia oil_ + 3 SD_100% chia oil_ (8)

## 3. Results and Discussion

**Figure 1:**
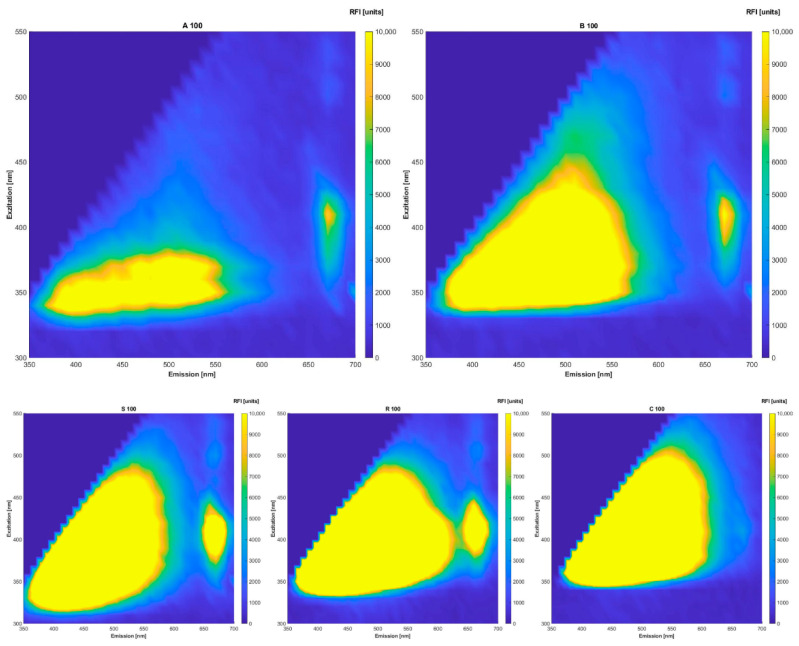
Fluorescence spectra of native oils, A: chia, B: chia, S: sunflower, R: rape seed, C: corn.

The native oils could easily be distinguished by their fluorescence spectra (Figure 1). All of the oils differ in intensities and slight intensity regions. It was assumed that the best results would be obtained through fluorescence spectra evaluation. The visible peaks can be assigned to pigments of groups belonging to NADH, tocopherols, riboflavin (emission 524 nm), oxidation products of oil ingredients e.g., vitamin E derivates at 525 nm emission and chlorophyll at excitation 405 nm and emission 670 nm [10,11,12,33,34,35]. However, the oils were not prepared in a special way or measured in a solvent; therefore, the ranges might have shifted and/or the intensities might be lower. Since we work with raw materials that are subject to natural variations, it is definitely possible that the spectra of two oils are not one in the same. The fluorescence spectra of the chia oils show the same intensity regions. Overall, all of the oils examined show higher intensities in the regions of carotenoids, tocopherols, polyphenols and chlorophylls. Lower intensities in the regions of 350 nm excitation and 400 nm to 450 nm emission indicate the presence of oxidation products formed during oil ageing. Observing Figure 1 in-depth, it is obvious that the intensities of the oils used for adulteration (sunflower, rape seed and corn) have higher intensities in the respective regions.

**Figure 2:**
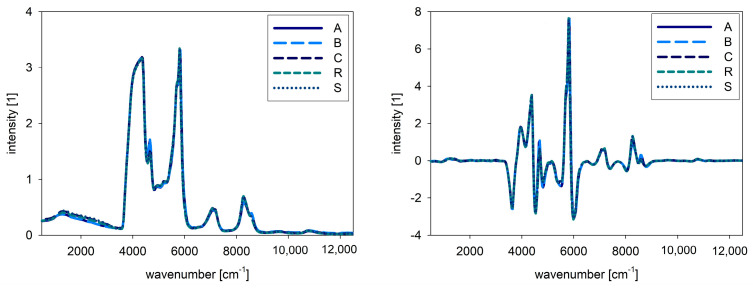
NIR spectra of native oils, A: chia, B: chia, R: rape seed, S: sunflower, C: corn, raw spectra (**left**), pre-processed spectra (**right**).

**Figure 3:**
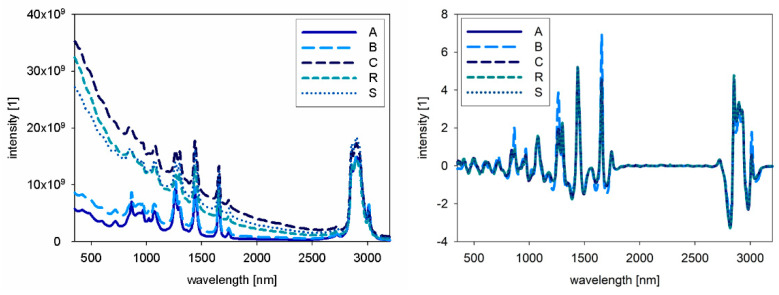
Raman spectra of native oils, A: chia, B: chia, R: rape seed, S: sunflower, C: corn, raw spectra (**left**), pre-processed spectra (**right**).

**Figure 4:**
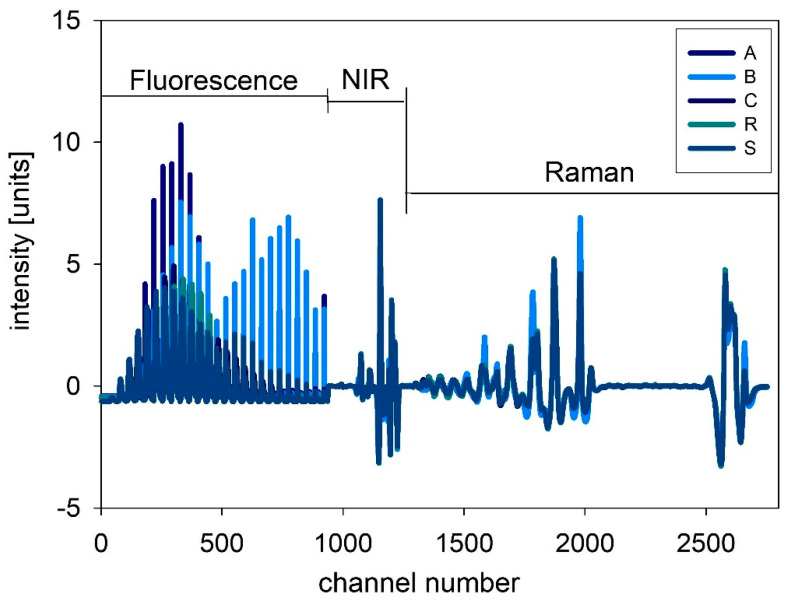
Combination of all spectra for evaluation.

For NIR and Raman spectra, the native oil spectra are presented in Figure 2 and Figure 3. The left side shows the raw spectra, whereas the right side shows the pre-processed spectra. For the combined evaluation, the fluorescence spectra were also pre-processed by SNV, and therefore the intensities are comparable. For NIR spectra, no big differences between the samples are obvious, but in the Raman spectra different intensities for the samples are visible. In Figure 4, the combined spectra of all native oils are presented. The spectra of A and B show differences compared to the other oils.

**Figure 5:**
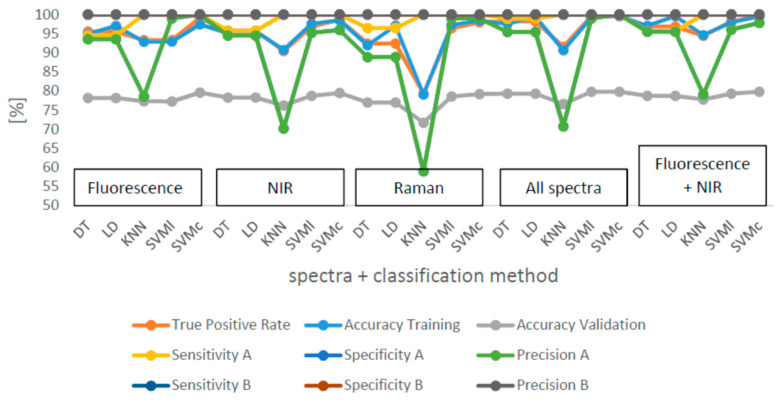
Median of 10 runs for TPR, accuracy of training and validation, sensitivity, specificity and precision of the classification for all evaluated spectra and combined variations as well as different classification methods; DT: decision tree, LD: linear discriminant analysis, KNN: nearest neighbour classification, SVMl and SVMc: support vector machines linear and cubic.

Mean values and standard deviations for the ten classification runs can be found in Table 4 and Table 5. The best results for the classification were obtained with a TPR of 99.7% for the classification with SVMc and the combination of all of the spectra together (Table 5). The combination of fluorescence and NIR spectra were classified with a TPR of 99.5% with SVMc, and SVMc is also the best classification method for all single spectra. The medians for the TPR, sensitivity, specificity and accuracy of the classification are presented in Figure 5. The median TPR is over 90% for most of the calculations. As usual, the training accuracies are, with exceptions, all over 90%, higher than the validation accuracies which were between 71% and 79.9%. The sensitivity as well as the specificity were over 90% for all of the samples. However, B was better detected. The precision was around 100% for pure B samples whereas for A, the precision was poor with 54.2 ± 3% for the Raman spectra classification by KNN. The precision for adulterated samples was over 90%. It is obvious that KNN results in the poorest classification results for A and B as well as for all measured spectra and their combinations. Adulterations for A were incorrectly classified.

**Figure 6:**
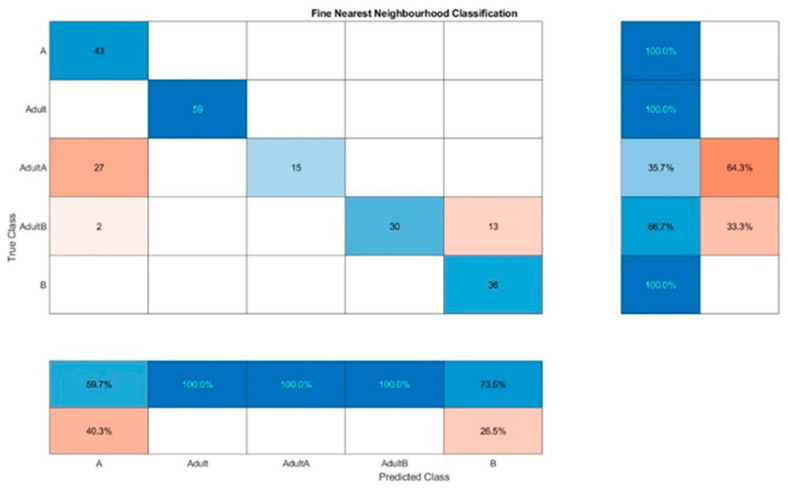
Confusion matrix for the oil classification by KNN out of the Raman spectra.

**Figure 7:**
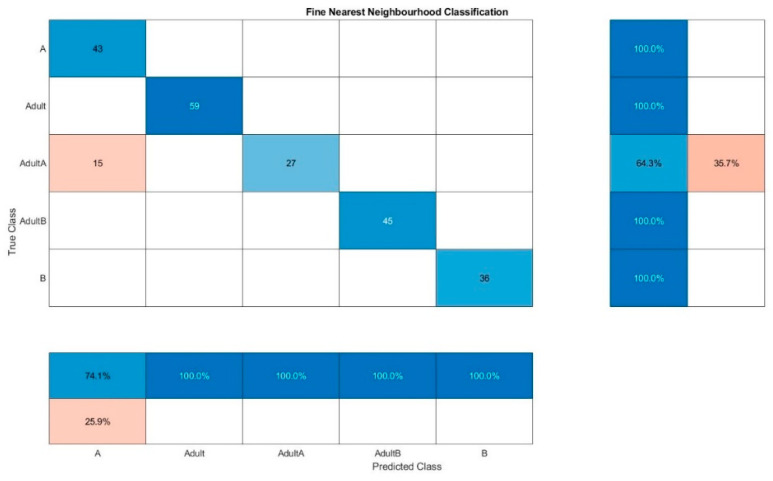
Confusion matrix for the oil classification by KNN out of the combined evaluation of the fluorescence and NIR spectra.

**Figure 8:**
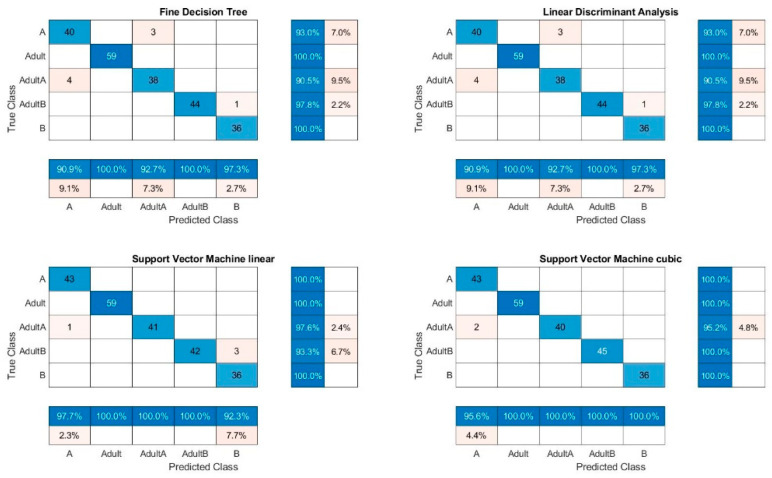
Confusion matrix for the oil classification by DT, LDA, SVMl & SVMc out of the combined evaluation of the fluorescence and NIR spectra.

For Raman spectra evaluation, KNN resulted in a false classification of 64.3% for Adult A and a false classification of 33.3% for Adult B for one out of ten classifications (Figure 6). The same classification method leads to the combined evaluation of fluorescence and NIR spectra (Figure 7) to only a false classification of 35.7% of Adult A, which indicated that somehow the adulteration samples of chia oil A are more difficult to detect in general. The best results were obtained for the combined evaluation of fluorescence and NIR spectra, the confusion matrix of one classification run is presented in Figure 8. The wrong classifications are more or less equally distributed over all samples and remain below 10%. A successful classification is hence possible for 5 classes. KNN does not seem to be sufficient for these classification processes.

The presented method was capable of identifying most of the samples in the validation trial. It is a fast method which is easy to use after a calibration. The quantification of other compounds in the oil might also be possible with this method but this was not the focus of this study. The time-saving after the calibration of a spectroscopic method is around 2 to 3 times faster [36]. This underlines the necessity of the validation, which was successfully performed in this study.

The best results of the PLSR are presented in Table 6. The coefficients of determination are above 0.95 for all samples. Given the fact that the extreme points (the native oils) could be distinguished quite easily, this is not surprising.

The RMSEP_range_ values are more interesting; they were, with one exception, all below 5%. For the regression of samples with chia oil A, the best results were obtained with NIR spectra. For B, the best results were obtained with combined spectra. The highest error, corrected to the range of the considered samples (A, B, R, S, C), was RMSEP_range_ = 10% for the evaluated Raman spectra alone, the lowest 1.3% for the combined evaluation of the spectra. The determination of the detection limit was not suitable for fluorescence spectra, as the smallest is 6.1%. However, for NIR (4.4%) and Raman, lower detection limits were obtained. It was found to be best with 3% of the spectra obtained with chia oil A adulterated with sunflower oil S for the combined spectra evaluation. The best result for combined spectra evaluation for chia oil B was also obtained with S as adulteration oil with a detection limit of 4.1%.

As can be seen in Table 7, for the Kenyan chia oils the RMSEP_range_ was between 0.6% and 16.7%. The detection limit varied according to the adulteration oil and it was better for the combined evaluations of the spectra. The measurements are regarded as unrepresentative because only a limited amount of sample was present. The detection limits were low (0.7/0.8 for Raman of U and Y), but the models had high RMSEP_ranges_, so the reliability of these results is questionable.

For this study, two Mexican chia oils and six Kenyan chia oils were evaluated. Therefore, the range within this study is higher than in the study presented by Rodríguez et al. [15]. The comparison is difficult as the methods and the study designs were different and it is not clear how they calculated their RMSEP. Here, six different classification methods were evaluated and a PLSR regression was performed to get an idea of the amount of adulteration and, furthermore the RMSEP_ranges_ were quite low in this study. The combination of all of the spectra was beneficial for the RMSEP_range_ and the PLSR as the range is here between 1.3% and 2.3%. This is better as presented by Rodríguez et al. [15] for the FT-IR analysis by SIMCA and OC-P-PLS. The comparison of the RMSEPs for the adulterated samples with A and B shows that, with one exception, the presented PLSR method is better than the other method, because the range of the RMSEP_range_ was between 1.3% and 4.8%. The classification sensitivity and specificity depended on the classification method which was sometimes lower, but mostly higher or at the same level. Oil B was easier to detect. However, it is difficult to compare the methods point by point, as the calculation of the RMSEP might be different as our RMSEP is standardized to the measurement range. For the Kenyan samples, the sample size was limited and the results might therefore be unrepresentative, but it proves the method working.

## 4. Conclusions

The aim of the study was to answer following questions. Is it possible to detect chia oil adulteration by spectroscopic analysis of the oils? Is it possible to identify the adulteration oil? Is it possible to determine the amount of adulteration? The presented results suggest that it is possible to distinguish between different oils by fluorescence, NIR and Raman spectroscopy. It is possible to detect adulterations of chia oils and to distinguish between different adulterations. Here, adulterations of chia oils by rapeseed, sunflower and corn oil were identified with a median of 90% for the TPR. The training accuracies were over 90%, the sensitivity and specificity of the classifications were over 90% too. B was easier to detect, so the precision was around 100% and the adulterated samples were identified with a precision of 97%. All classification methods show good results, however SVM were the best. However, the classification by KNN is not suitable for this situation. The PLSR of A + B showed R^2^ over 0.95 for all models. The best RMSEP_range_ of chia oil A was obtained by NIR spectra evaluation whereas it was best for oil B by combined evaluation of all spectra. The worst RMSEP_range_ was obtained for Raman prediction of BC (10%), the best for combined spectra predicting AS (1.3%). For the Kenyan chia oils, the RMSEP_range_ was between 0.6% and 16.7%. However, only a small number of samples were measured. Detection limits varied according to the adulteration oil and were better for the combined evaluations of the spectra. It is also possible to identify the amount of adulteration, though less than 5% adulteration is difficult to identify. Further evaluations might lead to even better results, as there was not enough sample provided from the Kenyan oils. In conclusion, it is possible to identify adulterations from native samples by spectral analysis of the oils, depending on the adulteration oil. It is also better to combine all methods because a lower RMSEP_range_ can be obtained. The best results might be obtained with a classification by SVM, to identify if an adulteration took place, with a following PLSR of all combined spectra to quantify it.

## Figures and Tables

**Table 1:** T Nutritional values of the oil samples, for A and B (chia oils) per 100 g, for R, S and C per 100 mL.

Sample Name	Energy [kJ]	Energy [kcal]	Fat [g]	Saturated Fatty Acids [g]	Single Unsat. Fatty Acids [g]	Multiple Unsat. Fatty Acids [g]	Vitamin E [mg]
A (chia oil)	3700	900	100	10.1	7.8	82.1	-
B (chia oil)	3700	900	100	10.6	7.2	82.2	-
R (rapeseed)	3404	828	92	6.5	60	25.5	46
S (sunflower)	3404	828	92	10	28	54	30
C (corn)	3404	828	92	13	28	51	37

**Table 2:** Sample preparation and labelling for the spectroscopic analysis. A and B are the two Mexican chia oils, S is sunflower oil, R is rapeseed oil and C is corn oil. All values are mass percentages.

Samples		Materials				
		A	B	S	R	C
native oils	A100	100%				
	B100		100%			
	S100			100%		
	R100				100%	
	C100					100%
samples with A	AS90	90%		10%		
	AS95	95%		5%		
	AS98	98%		2%		
	AS99	99%		1%		
	AR90	90%			10%	
	AR95	95%			5%	
	AR98	98%			2%	
	AR99	99%			1%	
	AC90	90%				10%
	AC95	95%				5%
	AC98	98%				2%
	AC99	99%				1%
samples with B	BS90		90%	10%		
	BS95		95%	5%		
	BS98		98%	2%		
	BS99		99%	1%		
	BR90		90%		10%	
	BR95		95%		5%	
	BR98		98%		2%	
	BR99		99%		1%	
	BC90		90%			10%
	BC95		95%			5%
	BC98		98%			2%
	BC99		99%			1%
additional combinations	RS50			50%	50%	
	RC50				50%	50%
	SC50			50%		50%
	AS50	50%		50%		
	AR50	50%			50%	
	AC50	50%				50%
	BS50		50%	50%		
	BR50		50%		50%	
	BC50		50%			50%

**Table 3:** Sample preparation for the additional Kenyan chia oil samples (U–Z) that were adulterated with sunflower oil (S). Samples indicated with * were prepared only once, the others were prepared two times.

Samples	Materials						
	U	V	W	X	Y	Z	S
U100 *	100%						
V100		100%					
W100			100%				
X100 *				100%			
Y100					100%		
Z100						100%	
US90 *	90%						10%
US50 *	50%						50%
VS90		90%					10%
VS50		50%					50%
WS50 *			50%				50%
XS90 *				90%			10%
XS50				50%			50%
YS90					90%		10%
YS50					50%		50%
ZS90						90%	10%
ZS50						50%	50%

* All together 142 samples are used for spectroscopic measurement.

**Table 4:** Results of the classification of samples with single spectra; Means and standard deviations of 10 classification runs.

**Fluorescence**	**1 Tree**	**2 LD**	**3 KNN**	**4 SVMl**	**5 SVMc**
TPR	94.9 ± 1.6	94.9 ± 1.6	93.2 ± 1.4	92.8 ± 1	98.1 ± 2
Accuracy training	95 ± 0.7	97.1 ± 0.5	92.9 ± 0.4	92.9 ± 0.4	97.5 ± 0.7
Accuracy validation	78 ± 0.6	78 ± 0.6	77.3 ± 0.6	77.1 ± 0.4	79.3 ± 0.8
Sensitivity A	94.6 ± 2.9	94.6 ± 2.9	100 ± 0	100 ± 0	100 ± 0
Sensitivity B	100 ± 0	100 ± 0	100 ± 0	100 ± 0	100 ± 0
Specificity A	97.9 ± 1.1	97.9 ± 1.1	93.3 ± 1.8	99.6 ± 0.5	99.8 ± 0.3
Specificity B	100 ± 0	100 ± 0	100 ± 0	100 ± 0	100 ± 0
Precision A	91.2 ± 4.9	91.2 ± 4.9	78.1 ± 6.3	98.1 ± 2.7	99.3 ± 1.2
Precision B	100 ± 0	100 ± 0	100 ± 0	100 ± 0	100 ± 0
**NIR**	**6 tree**	**7 LD**	**8 KNN**	**9 SVMl**	**10 SVMc**
TPR	95.8 ± 2.2	95.8 ± 2.2	90.2 ± 1.8	97.2 ± 1.3	98.4 ± 0.7
Accuracy training	95.3 ± 0.5	95.5 ± 0.8	90.7 ± 0.3	97.6 ± 0.4	98.5 ± 0.3
Accuracy validation	78.3 ± 0.9	78.3 ± 0.9	76.1 ± 0.7	78.9 ± 0.5	79.4 ± 0.3
Sensitivity A	95.5 ± 2.9	95.5 ± 2.9	100 ± 0	100 ± 0	100 ± 0
Sensitivity B	99.5 ± 1.1	99.5 ± 1.1	100 ± 0	100 ± 0	100 ± 0
Specificity A	98.7 ± 0.9	98.7 ± 0.9	90.2 ± 1.7	98.6 ± 0.9	99 ± 0.6
Specificity B	100 ± 0	100 ± 0	100 ± 0	100 ± 0	100 ± 0
Precision A	94.7 ± 3.1	94.7 ± 3.1	71 ± 5.4	94.4 ± 3.2	96.1 ± 2.2
Precision B	100 ± 0	100 ± 0	100 ± 0	100 ± 0	100 ± 0
**Raman**	**11 tree**	**12 LD**	**13 KNN**	**14 SVMl**	**15 SVMc**
TPR	92.2 ± 2.5	92.2 ± 2.5	79.6 ± 2.6	96.4 ± 0.7	98 ± 0.4
Accuracy training	92.3 ± 1.2	97 ± 0.3	79 ± 0.6	97.3 ± 0.3	98.4 ± 0.2
Accuracy validation	76.9 ± 1	76.9 ± 1	71.8 ± 1	78.6 ± 0.3	79.2 ± 0.2
Sensitivity A	96 ± 3.6	96 ± 3.6	100 ± 0	100 ± 0	100 ± 0
Sensitivity B	98.5 ± 2	98.5 ± 2	100 ± 0	100 ± 0	100 ± 0
Specificity A	96.5 ± 2.1	96.5 ± 2.1	81.5 ± 2.9	99.5 ± 0.5	99.7 ± 0.3
Specificity B	99.9 ± 0.2	99.9 ± 0.2	100 ± 0	100 ± 0	100 ± 0
Precision A	87.1 ± 7.1	87.1 ± 7.1	56.7 ± 5.8	97.9 ± 2.4	98.8 ± 1.3
Precision B	99.8 ± 0.8	99.8 ± 0.8	100 ± 0	100 ± 0	100 ± 0

**Table 5:** Results of the classification of samples with combinations of spectra; means and standard deviations of 10 classification runs.

**Fluo + NIR + Raman**	**16 Tree**	**17 LD**	**18 KNN**	**19 SVMl**	**20 SVMc**
TPR	98.1 ± 0.6	98.1 ± 0.6	91.2 ± 2	99.2 ± 0.4	99.7 ± 0.3
Accuracy training	97.5 ± 0.6	99.4 ± 0.1	90.7 ± 0.3	99.2 ± 0.2	99.6 ± 0.2
Accuracy validation	79.2 ± 0.3	79.2 ± 0.3	76.5 ± 0.8	79.7 ± 0.2	79.9 ± 0.1
Sensitivity A	98.3 ± 2	98.3 ± 2	100 ± 0	100 ± 0	100 ± 0
Sensitivity B	99.7 ± 0.9	99.7 ± 0.9	100 ± 0	100 ± 0	100 ± 0
Specificity A	98.8 ± 0.5	98.8 ± 0.5	89.7 ± 1.9	99.5 ± 0.6	99.8 ± 0.3
Specificity B	99.9 ± 0.4	99.9 ± 0.4	100 ± 0	100 ± 0	100 ± 0
Precision A	95.2 ± 2.2	95.2 ± 2.2	70 ± 5.4	97.9 ± 2.9	99.3 ± 1.2
Precision B	99.7 ± 1	99.7 ± 1	100 ± 0	100 ± 0	100 ± 0
**Fluo + NIR**	**21 tree**	**22 LD**	**23 KNN**	**24 SVMl**	**25 SVMc**
TPR	97.1 ± 1	97.1 ± 1	94.5 ± 1.1	98.4 ± 1.1	99.5 ± 0.5
Accuracy training	97.1 ± 0.7	99.5 ± 0.2	94.5 ± 0.3	98 ± 0.3	99.5 ± 0.1
Accuracy validation	78.8 ± 0.4	78.8 ± 0.4	77.8 ± 0.4	79.3 ± 0.4	79.8 ± 0.2
Sensitivity A	94.6 ± 3.8	94.6 ± 3.8	100 ± 0	100 ± 0	100 ± 0
Sensitivity B	99.7 ± 0.9	99.7 ± 0.9	100 ± 0	100 ± 0	100 ± 0
Specificity A	99 ± 0.7	99 ± 0.7	93.6 ± 1.4	99 ± 0.8	99.3 ± 0.5
Specificity B	100 ± 0	100 ± 0	100 ± 0	100 ± 0	100 ± 0
Precision A	95.7 ± 3.2	95.7 ± 3.2	78.8 ± 5.1	95.7 ± 3.5	97.2 ± 2.6
Precision B	100 ± 0	100 ± 0	100 ± 0	100 ± 0	100 ± 0

**Table 6:** Results of the best PLSR predictions for the single oils A and B with single evaluations of the adulteration oils R, S, and C and the combination of all adulterations with the samples all separately for all methods and the combination of all methods.

	Oil	R^2^	RMSEPrange	Detection Limit [%]
Fluorescence with Preprocessing	AC	0.994	2.7	8.3
	AR	0.988	3.8	8.6
	AS	0.993	2.9	7.2
	A all	0.991	3.4	8.7
	BC	0.992	3.2	6.1
	BR	0.992	3.1	17.9
	BS	0.983	4.7	18.6
	B all	0.990	3.6	15.4
Fluorescence without Preprocessing	AC	0.994	2.7	10.0
	AR	0.988	3.9	9.4
	AS	0.993	2.9	7.4
	A all	0.991	3.4	7.8
	BC	0.991	3.3	7.6
	BR	0.992	3.1	17.9
	BS	0.981	4.8	23.5
	B all	0.991	3.5	10.5
NIR	AC	0.997	2.1	6.0
	AR	0.995	2.4	7.4
	AS	0.997	2.1	4.5
	A all	0.995	2.5	6.3
	BC	0.997	2.0	6.6
	BR	0.997	1.9	5.8
	BS	0.997	2.0	4.4
	B all	0.996	2.3	6.2
Raman	AC	0.992	3.2	5.5
	AR	0.993	3.0	9.0
	AS	0.994	2.7	5.3
	A all	0.984	4.6	11.2
	BC	0.920	10.0	18.1
	BR	0.986	4.1	11.6
	BS	0.932	9.2	7.0
	B all	0.938	8.9	12.0
Combined	AC	0.998	1.7	3.9
	AR	0.996	2.3	7.4
	AS	0.999	1.3	3.0
	A all	0.997	2.1	5.8
	BC	0.997	1.9	6.9
	BR	0.998	1.6	6.8
	BS	0.998	1.5	4.1
	B all	0.997	1.8	4.3

**Table 7:** Results of the best PLSR predictions for the single chia oils U, V, W, X, Y and Z with single evaluations of the adulteration oil S separately for all methods and the combination of all methods.

	Oil	R^2^	RMSEPrange	Detection Limit [%]
Fluorescence with Preprocessing	U	0.999	1.5	3.8
	V	0.995	3.0	5.3
	W	0.999	1.5	3.1
	X	0.994	3.1	2.5
	Y	0.993	3.5	4.7
	Z	0.997	2.3	6.7
Fluorescence without Preprocessing	U	0.999	1.3	3.5
	V	0.995	3.1	5.0
	W	0.999	1.6	2.9
	X	0.994	3.1	2.6
	Y	0.993	3.5	4.6
	Z	0.997	2.1	7.8
NIR	U	1.000	0.6	1.6
	V	0.995	3.1	11.3
	W	0.998	1.9	5.6
	X	0.999	1.0	2.6
	Y	0.999	1.3	2.2
	Z	0.999	1.1	3.0
Raman	U	0.910	12.8	0.7
	V	0.838	16.7	52.0
	W	0.872	16.1	57.8
	X	0.889	13.2	2.2
	Y	0.865	14.9	0.8
	Z	0.838	16.7	52.0
Combined	U	1.000	0.6	1.6
	V	0.996	2.7	8.0
	W	0.999	1.4	2.7
	X	1.000	0.6	1.7
	Y	0.999	1.3	3.3
	Z	0.999	1.1	3.8
